# An Iranian Congenital Adrenal Hypoplasia Patient with Elevated Testosterone in Infancy due to a Novel Pathogenic Frameshift Variant in *NR0B1*

**DOI:** 10.1155/2021/4367028

**Published:** 2021-12-13

**Authors:** Samira Kalayinia, Saeed Talebi, Mohammad Miryounesi, Peymaneh Sarkhail, Nejat Mahdieh

**Affiliations:** ^1^Cardiogenetic Research Center, Rajaie Cardiovascular Medical and Research Center, Iran University of Medical Sciences, Tehran, Iran; ^2^Department of Medical Genetics, Faculty of Medicine, Iran University of Medical Sciences, Tehran, Iran; ^3^Department of Medical Genetics, Faculty of Medicine, Shahid Beheshti University of Medical Sciences, Tehran, Iran; ^4^Pediatric Department, Atieh Hospital, Tehran, Iran; ^5^Growth and Development Research Center, Tehran University of Medical Sciences, Tehran, Iran

## Abstract

X-linked congenital adrenal hypoplasia due to *NR0B1* mutation is characterized by hypogonadotropic hypogonadism (HH) and infertility. Here, we describe a novel pathogenic frameshift variant in *NR0B1* associated with congenital adrenal hypoplasia by whole exome sequencing in an Iranian case with high level of testosterone. Clinical evaluations and pedigree drawing were performed. Point mutations, gene conversions, and large deletions of the *CYP21A2* gene were checked. WES and segregation analyses were conducted. In silico analysis was also performed for the novel variant. The ACTH, 17-hydroxy progesterone c, and DHEA sulfate values were elevated up to 624.6 pg/mL, 8.6 pmol/L, and 17.8UMOL/L, respectively. No mutation was found in the CYP21A2 gene. WES identified a novel hemizygous frameshift insertion c.218_219insACCA: p.His73GlnfsTer41 variant in the *NR0B1* gene with a pathogenic effect according to ACMG criteria. Genetic testing is helpful for differential diagnosis in primary adrenal insufficiency disorders. *NR0B1* may be a common cause of congenital adrenal hypoplasia in our population.

## 1. Introduction

*NR0B1* mutation leads to the congenital adrenal hypoplasia (CAP) as a rare X-linked disorder with an estimated prevalence of 1 : 140,000–1 : 1,200,000 in a general population [1]. X-linked congenital adrenal hypoplasia is usually characterized by adrenal insufficiency at ages from infancy to early childhood and hypogonadotropic hypogonadism (HHG) and infertility at later ages in males [2]. This gene encodes a transcription factor named DAX1 which is a member of the nuclear receptor superfamily. It has an important role in development of the adrenal and reproductive function and axes [3, 4]. *NR0B1* mutations usually result in primary adrenal insufficiency and hypogonadotropic hypogonadism; however, there are some reports of patients with elevated levels of testosterone [5, 6]. The severity of clinical characteristics including degree and onset of adrenal insufficiency due to different mutations of *NR0B1* has been shown to vary in individuals with adrenal insufficiency and reproductive phenotypes [1, 7]. Mild phenotypes usually result from residual activity of the mutant protein [8].

More than two hundreds variants have been reported in the *NR0B1* gene (http://www.hgmd.cf.ac.uk). About one-third of its mutations are small indels as frameshift variants. Here, we describe a novel frameshift variant in an Iranian infant with high level of testosterone.

## 2. Materials and Methods

### 2.1. Sampling and Whole Exome Sequencing

A small family with a CAH-affected son was referred to our center. The patient had been screened for all exons and intron boundaries of the *CYP21A2* gene previously, and no pathogenic variants were detected. After signing the informed consent, approved by the RHC Ethics Committee, DNA was extracted from obtained blood of the family members (healthy/patient). Exome was captured by using anAgilent SureSelect Exome Capture kit (Agilent Inc., Santa Clara, California, USA). Then, the sequencing of the enriched exon libraries was performed on the Illumina HiSeq 4000 (Macrogen Inc., Seoul, South Korea).

### 2.2. Read Mapping, Snp/Indel Calling, and Filtering

The sequencing reads were aligned to the human genome reference (GRCh37 build) by the BWA (v07.17) tool [9]. The quality of exome data mapping to the reference genome was 98.8%, and the target region coverage was 99%. Next, snp/indel was called applying the GATK (v4.1.4.1) tool with the result file of mapping (BAM). The mark and remove duplicates were performed by SAMtools (in GATK package) [10]. Then, recalibration and snp/indel calling were performed. The confident variants were filtered and prioritized due to minor allele frequency (MAF >0.05) of 1000 genome, gnomAD (v2.1.1), and ExAc [11] databases.

### 2.3. PCR and Sanger Sequencing of NR0B1

The variant of the *NR0B1* gene was sequenced applying the PCR and Sanger sequencing method. The primer pairs, i.e., 5´-ACTGGGCAGAACTGGGCTAC-3´ and 5´-GCGCTTGATTTGTGCTCGT-3´, were designed and validated using Primer3 (v.04.0) (http://bioinfo.ut.ee/primer3-0.4.0/) and BLAST (https://www.ncbi.nlm.nih.gov/tools/primer-blast/index.cgi?LINK_LOC=BlastHome). The Sanger sequencing was performed using the BigDye Terminator v3.1 Cycle Sequencing Kit (Life Technologies; Thermo Fisher Scientific, Shanghai, China) on ABI Sequencer 3500XL PE (Applied Biosystems, CA, USA).

### 2.4. Homology Modeling and Bioinformatics Analysis

Homology modeling was used to obtain a 3D structural model of NR0B1 protein, due to the lack of crystal structure for this protein and evaluation of the mutation effect on structure and function. The *NR0B1* sequence was retrieved from the UniProt database (UniProt ID: P51843). The SWISS-MODEL server (https://swissmodel.expasy.org/) [12] was applied for homology modeling. The protein FASTA sequence was submitted as an input, without assigning any restraints and templates. The SWISS-MODEL models structures of target protein and comprises conserved ligands by using BLAST and HHblits. It indicates the model quality estimation per residue based on a QMEAN score, and CAMEO monitors the accuracy of SWISS-MODEL, overlay. In addition, bioinformatics analysis was performed applying algorithms to evaluate the pathogenicity of detect variants. The prediction tools such as CADD, SIFT, Polyphen2, Provean, fathmm, and GERP^++^ were used. The variant that was interpreted to be pathogenic in at least 4 algorithms was considered for confirmation/segregation analysis.

## 3. Results

### 3.1. Clinical Characteristics

**Figure 1:**
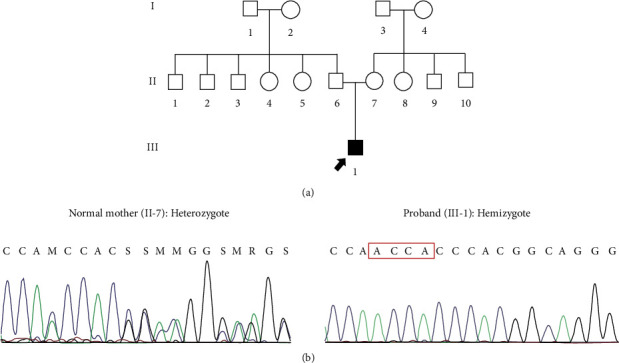
(a) Pedigree of the studied family with the patient denoted in black. (b) DNA sequence chromatograms indicate the inherited c.218_219insACCA: p.His73GlnfsTer41 variant in *NR0B1*, encoding the nuclear receptor subfamily 0 group B member 1.

The patient (III-1) was 9-month-old Iranian boy from a first cousin couple. There was no history of medical problems in the pedigree (Figure 1(a)). The infant was introduced to our center at the same age 9 months, with the diagnosis of CAH for genetic testing. The patient's history since birth was as follows: he was born by cesarean section, the birth weight and length were 3.25 kg and 53 cm, respectively, and blood sugar was at a normal level since the first days of life. The hyperbilirubinemia was observed in the second day after birth, and four days of phototherapy treatment was performed. Several days after discharge, he was admitted again due to vomiting and jaundice (total bilirubin = 14 mg/dL). Electrolytes were evaluated in the first step of workup that indicated hyponatremia (sodium of 125 mmol/L), hyperkalemia (potassium of 7.5 mmol/L), and metabolic acidosis (Table 1). The medical record was significant for hyponatremia and hypokalemia. Despite the treatment with sodium chloride and fludrocortisone, hyponatremia continued. Failure to thrive, poor feeding, vomiting, and seizures have been reported for this infant; however, there were not any dysmorphic genitalia and features. The ACTH, 17-hydroxy progesterone c, and DHEA sulfate values were elevated up to 624.6 pg/mL, 8.6 pmol/L, and 17.8 UMOL/L, respectively. Our patient showed a high level of testosterone.

### 3.2. Genetic Investigations

After filtering the WES data, the novel hemizygous frameshift insertion c.218_219insACCA: p.His73GlnfsTer41, in exon 1 of the *NR0B1* gene, was identified which is probably responsible for CAH in this family. The c.218_219insACCA was confirmed and segregated in the studied proband as well as his mother by using PCR and Sanger sequencing. According to the segregation analysis result, CAH inherited in this family with X-linked recessive pattern (Figure 1).

**Figure 2:**
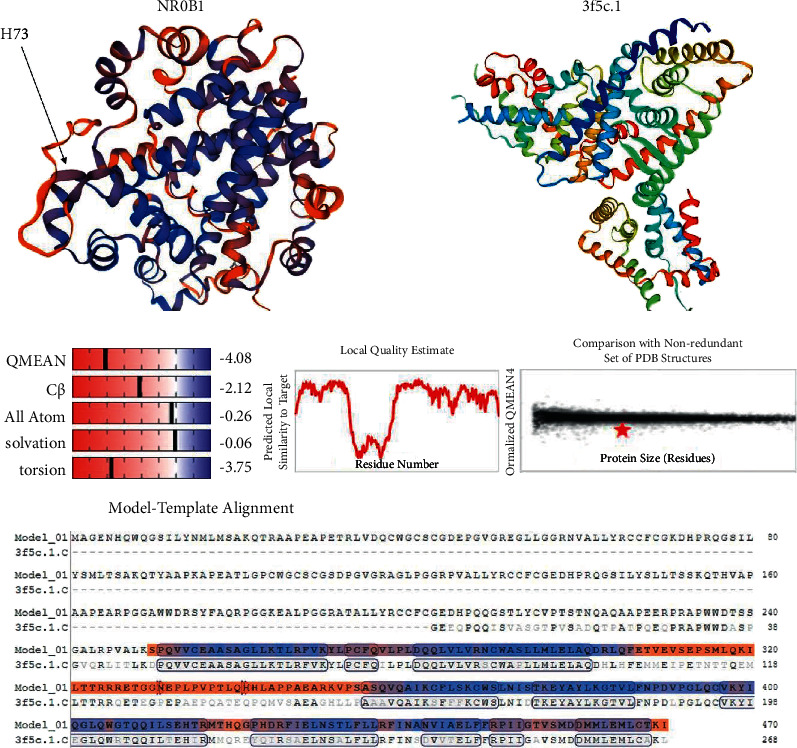
The SWISS-MODEL templates library (3f5c.1) and homology modeling of NR0B1.

The best model of NR0B1 was totally designed using 3f5c.1, due to SWISS-MODEL templates library, as the first ranked template with 79% identity. This structure had a QMEAN score of −4.08 that indicates the model is more reliable with low local error score. Due to the complete destruction of NR0B1 protein resulting from p.His73GlnfsTer41 variant, no mutant structure model was obtained (Figure 2). Given American College of Medical Genetics and Genomics 2015 (ACMG) [14], the c.218_219insACCA is determined as a pathogenic variant, i.e., criteria: PVS1, PM2, PP4, PP1, and PP3. The frameshift insertion variant was supported as the cause of disease by CADD, SIFT, Polyphen2, Provean, Fathmm, and GERP^++^.

## 4. Discussion

Primary adrenal insufficiency (PAI) is an etiologically heterogeneous group of disorders which could be potentially life threatening. As an acute onset form of PAI, X-linked congenital adrenal hypoplasia (CAP) is characterized by hypogonadotropic hypogonadism (HH) and infertility. *NR0B1* mutation leads to CAP. Here, we describe a novel pathogenic frameshift variant in NR0B1 associated with congenital adrenal hypoplasia by whole exome sequencing in an Iranian case for the first time.

DAX1 encoded by *NR0B1* has an essential function during the development of the adrenal cortex and reproductive axes. In CAP patients, laboratory findings show electrolyte disturbances, low androstenedione and low testosterone levels, an increased plasma renin activity, and decreased aldosterone level. More than ten patients with NR0B1 mutations have been reported, but their testosterone levels have been elevated [6]. Our patient showed a high level of testosterone. Premature pubarche, thus, is expected to be observed in our patient in near future of his life. Choosing an appropriate preventive approach for treatment is a crucial step.

Of 277 *NR0B1* mutations, 108 are frameshift. The c.218_219insACCA as a small insertion leads to a frameshift variant, p.His73GlnfsX41, which is categorized as a pathogenic variant based on ACMG guidelines. As expected, this variant produced a truncated protein with abnormal function. The majority of frameshift variants show their phenotype at earlier ages.

There are many reports about congenital adrenal hyperplasia [15, 16], but this case is the first patient with CAP from Iran. Some mutations have a unique pattern of distribution in some populations; e.g., some of the *CYP21A2* mutations are common in many cohorts, but some of them are unique [16]. The c.218_219insACCA may have an ancestor in our population, so further studies are recommended to clarify this hypothesis.

In conclusion, genetic testing is helpful for differential diagnosis of PAI because variability of phenotypes among patients may lead to a wrong clinical diagnosis. *NR0B1* may be a common cause of CAP among Iranian subpopulations. Early diagnosis of this disorder could be helpful in treatment.

## Figures and Tables

**Table 1:** Biochemical analyses of the CAH patient blood

Parameters (unit)		Measured values in the patient	Reference values [13]
Serum	Blood sugar (mg/dL)	81	<140
	Sodium (mmol/L)	138	135–146
	Potassium (mmol/L)	4.4	4.1–5.3

Plasma	ACTH (pg/mL)	**624.6**	7.2–63
	Renin supine (mcIU/mL)	140	4.2–59.7

Serum	17-Hydroxy progesterone c (pmol/L)	**8.6**	0.2–2.9
	Testosterone (ng/ml)	3.1	0.12–0.2
	DHEA sulfate (UMOL/L)	**17.8**	0–6.7
	Androstenedione (ng/mL)	0.05	0.08–2.5
	Cortisol (*µ*g/dL)	5.3	3.7–19.4

## Data Availability

The data used to support the findings of this study are available from the corresponding author upon request.
